# Topological momentum skyrmions in Mie scattering fields

**DOI:** 10.1515/nanoph-2025-0071

**Published:** 2025-05-05

**Authors:** Peiyang Chen, Kai Xiang Lee, Tim Colin Meiler, Yijie Shen

**Affiliations:** Centre for Disruptive Photonic Technologies, School of Physical and Mathematical Sciences, 54761 Nanyang Technological University, Singapore 637371, Singapore; School of Physics and Astronomy, Shanghai Jiao Tong University, Shanghai 200240, China; Zhiyuan College, Shanghai Jiao Tong University, Shanghai 200240, China; Centre for Quantum Technologies, National University of Singapore, Singapore 117543, Singapore; Institute of Materials Research and Engineering, A*STAR (Agency for Science Technology and Research), Singapore 138634, Singapore; School of Electrical and Electronic Engineering, 54761 Nanyang Technological University, Singapore 639798, Singapore

**Keywords:** optical skyrmion, nanophotonics, Mie scattering, topology, momentum of light

## Abstract

How topologies play a role in light–matter interaction is of great interest in control and transfer of topologically-protected structures. These topological structures such as skyrmions and merons have not yet been found in canonical momentum fields, which are fundamental in mechanical transfer between optical and matter fields. Here, we reveal the universality of generating skyrmionic structures in the canonical momentum of light in multipole Mie scattering fields. We demonstrate the distinct topological stability of canonical momentum skyrmions and merons, and compare with well-studied Poynting vector and optical spin fields. The study of these fields allow for a clean and direct approach to measuring and quantifying energetic structures in optical fields, through observable radiation pressure. Our work lays the foundation for exploring new topologically nontrivial phenomena in optical forces, metamaterial design, and light–matter interaction.

## 1 Introduction

Skyrmions are topological nontrivial vector textures, first proposed in particle physics as a low-energy nucleon model [1]. Over subsequent decades, skyrmions have been intensively studied in condensed matter domain and realized across a diverse range of systems, including atomic condensates [2], [3], liquid crystals [4], [5], [6], and chiral magnets [7], [8], [9]. In particular, skyrmions in materials have demonstrated their potential as novel topologically protected information carriers for large-density data storage [9], [10], [11]. Recently, optical structured vector fields have emerged as a rich and versatile platform for studying skyrmionic topologies as an analog to matter-based skyrmions [12], [13], and have demonstrated many practical applications in magnetic domain observation and displacement sensing [14], [15], [16]. These quasiparticle topologies of light have been realized in various basic vector associated with electromagnetic waves, such as electric and magnetic fields [17], [18], [19], [20], [21], optical spins [22], [23], [24], [25], polarization Stokes vectors [26], [27], [28], and Poynting vector fields [29]. However, one fundamental vector quantity of the electromagnetic field has thus far been overlooked – canonical momentum. Theoretically, canonical momentum is the mostly fundamental quantity to provide a clean and direct approach to measuring and quantifying energetic structures in optical fields, directly related to observable mechanical motion. The study of canonical momentum topological textures is urgently to be opened for the bases of topologically nontrivial light–matter interaction.

The momentum of light determines the energy transfer of the optical field as it propagates over free space and when it interacts with matter [30]. However, one must be careful in determining this particular quantity. One common method to quantify the energy flow (and thus momentum) of an optical field is the Poynting vector [31]. This quantity is a convenient measure that requires knowledge of the electric and magnetic fields to compute. However, the identification of this quantity with energy flow is not universal, with many irregularities and controversies raised since its conceptualization [32], [33], [34], [35]. In light–matter interactions, optical forces can arise from different physical phenomena. The canonical momentum density component of the energy flow is of particular interest [36], because it is collinear with the phase gradients of the optical field and is the dominant component of the radiation pressure force. This component provides a clean and direct approach for the measurement and quantification of energetic structures in optical fields [37] – with sufficiently small test particles or atoms, one can observe energetic structures at the sub-wavelength scale, below the diffraction limit [38], [39]. In particular, the theoretical studies of Poynting vector skyrmions have only recently emerged [29], [40], but they cannot unveil the role of topological textures in interaction with matter, as the Poynting momentum is an aggregate quantity in collinear case.

In this Letter, we theoretically demonstrate the formation of topological textures, including skyrmions and merons, in the canonical momentum vectors of a multipole scattering field. We show that the topology and helicity can be tuned by adjusting the phase difference of multipole sources and light polarization. We compare their topological dynamics with the topological textures of Poynting vector and optical spin. We also demonstrate their distinct topological stability against geometric defects in the scattering sources.

## 2 Theory and calculation

We first define key terms such as the Poynting vector **P** and kinetic momentum density **p** which are related in the following way:

(1) $$p=\frac{1}{c^{2}}P=\frac{1}{2c^{2}}R\left(E^{*}\times H\right)=p^{o}+p^{s}$$

where *c* is the speed of light, **E** and **H** are electric and magnetic fields, respectively. They can be split into two components, namely, the canonical momentum density **p** ^**o**^ and the virtual momentum density **p** ^**s**^, which arises from the linear momentum analog to the spin angular momentum density [41]. The former is defined as:

(2) $$p^{o}=\frac{1}{4\omega }I\left[\varepsilon E^{*}\cdot \left(\nabla \right)E+\mu H^{*}\cdot \left(\nabla \right)H\right]$$

where *ω* is the angular frequency, *ɛ* and *μ* represent dielectric and magnetic permeability of the propagation medium, respectively. Furthermore, it is related to the orbital angular momentum density **L** = **r** × **p** ^**o**^, while the spin part of the momentum density $p^{s}=\frac{1}{2}\nabla \times S$ is defined with respect to the spin angular momentum densities $S=\frac{1}{4\omega }I\left[\varepsilon \left(E^{*}\times E\right)+\mu \left(H^{*}\times H\right)\right]$ .

A topological configuration can be characterized by its topological charge $N=\frac{1}{4\pi }\iint_{\sigma }n\cdot \left(\partial_{x}n\times \partial_{y}n\right)dxdy=\iint_{\sigma }\rho \left(x,y\right)dxdy$ where *ρ*(*x*, *y*) represents the topological charge density of the vector field and *σ* traces the boundary of integration (Supplementary Section III). A skyrmion has a topological charge of *N* = 1 while a meron carries a fractional topological charge of *N* = 0.5.

Engineering skyrmions formed by momentum requires consideration of the symmetries within the physical domain. The key factor to constructing the skyrmionic configurations lies in breaking the symmetry along normal direction. In our study, we utilize the asymmetry of the far-field multipole radiation from Mie scattering.

The Mie solution to Maxwell’s equations describes the scattering of an electromagnetic plane wave by a spherical particle (Supplementary Section I). The contributions of the different scattered multipoles can be tuned precisely by engineering the properties of the particle [42], [43]. Mie scattering is a common approach for describing nanoparticle scattering and represents a simplified case of multipole scattering. The Mie coefficients *a*_*n*_ and *b*_*n*_ correspond to different orders *n* of multipole components. They can be independently tuned via a well-established approach by constructing a metasurface consisting of a periodic array of nanoparticles of identical shape with inversion symmetry, e.g., sphere, cylinder, cube [44]. Taking an array of cone-shaped nanoparticles as an example, it is possible to obtain multipole components with different proportions and phases by tuning the particle size and the wavelength of the incident light [45]. By utilizing these degrees of freedom, we show that one can tune the structure of the scattered radiation field to create skyrmions and merons in the far field.

**Figure 1:**
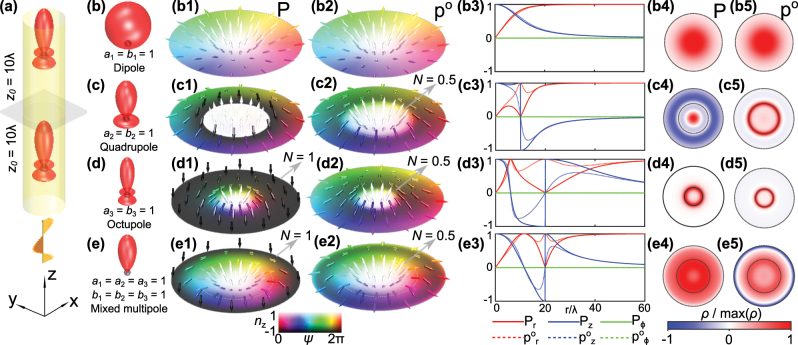
Momentum textures of scattered fields from two multipoles in the Kerker condition. (a) Schematic of the system. A light wave comes in from the bottom and is scattered by two particles symmetrically arranged around the examined plane. (b–e) Primary intensity lobes of the scattered radiation fields from multipoles. (b1–e1) Normalized Poynting momentum **P** textures host skyrmions with topological charge *N* = 1 for octupole (d1) and mixed multipole (e1). (b2–e2) Normalized canonical momentum **p** ^**o**^ texture shows merons with *N* = 0.5 for quadrupole (c2), octupole (d2) and mixed multipole (e2). The radius of all these texture plots is 2*z*_0_ = 20*λ*. (b3–e3) The change of normalized **p** and **p** ^**o**^ with respect to *r*, when the multipole source corresponds to (b–e), respectively. The colorbars indicate the direction of normalized vectors $\vec{n}$ , out-of plane component *n*_*z*_ and azimuth angle $\psi =arctan\frac{n_{y}}{n_{x}}$ . This colorbar is consistently used throughout the article to illustrate topological textures. (b4–e4) Normalized topological charge density of **P** textures. (b5–e5) Normalized topological charge density of **p** ^**o**^ textures. The radius of all these topological charge density plots is 2*z*_0_ = 20*λ*. The inner black circular line represents the meron boundary.

Consider a two-particle system uniformly illuminated by an incoming plane-wave along the optical axis, shown in Figure 1(a). The particles have identical scattering properties. We examine the scattered kinetic and canonical momenta in the plane equidistant from both particles. The particles are separated by 2*z*_0_ = 20*λ*. Finite radii of topological features such as skyrmions and merons scale proportional to the particle separation as long as the scattered radiation remains in the far field to avoid complications with evanescent components as well as inter-particle couplings, which is a key assumption in the multipole expansion.

## 3 Results

### 3.1 Typical topological configurations

In the following, we examine the symmetric case where the electric and magnetic field components have the same Mie scattering coefficients, which is known as the Kerker condition [46]. The Kerker condition is known for highly directive scattering into the far field. As forward scattering dominates over backscattering, the overlap of multipole radiation in the examined plane is inhomogeneous and different topological structures can be generated. We start with three simple yet representative cases: dipole, quadrupole and octupole.

When multipole sources are pure dipoles (*a*_1_ = *b*_1_ = 1), merons are formed in the kinetic and canonical momentum fields in the examined plane (Figure 1(b1) and (b2)). In this case, the meron boundary extends to infinity where their *z*-components vanishes (Figure 1(b3)).

For pure quadrupole sources (*a*_2_ = *b*_2_ = 1), a meron is realized in the canonical momentum field within a finite boundary *r* = 10*λ*. In contrast, the kinetic momentum field has a discontinuity at *r* = 10*λ* (Figure 1(c3)) due to vanishing electric and magnetic fields, so a topological invariant cannot be quantified. Similar meron textures appear in the pure even-ordered Kerker multipoles (quadrupoles, hexadecapoles, etc.) (Supplementary Section II).

In pure octupole radiation (*a*_3_ = *b*_3_ = 1), the kinetic momentum field hosts a skyrmion (Figure 1(d1)), whereas a meron is present in the canonical momentum field (Figure 1(d2)). The boundary of the skyrmion is realized at the radius *r* = 20*λ* where the Poynting vector field vanishes (Figure 1(d3)). Likewise, skyrmions and merons are realized for the pure odd-order Kerker multipoles except dipoles, e.g. octupoles, dotriacontapoles, and higher orders (Supplementary Section II).

Here, we explain the reasons for the formation of different topological textures in the kinetic and canonical momentum fields. The former is proportional to the Poynting vector, and we can discuss the appearance of the topological structures in the same context. In the examined plane, the Poynting vector texture arises from the superposition of the polar distribution of intensity lobes of the multipole scatterers. The scattering of Kerker particles is suppressed in the backward direction, causing **P** and **p** ^**o**^ to always be directed along the positive *z*-axis at the center of the textures in our system. In particular, even-order pure multipolar sources have an equal number of lobes in forward and backward radiation. This allows the Poynting vector field to undergo discontinuity radially before forming a complete skyrmion, where the electromagnetic field vanishes. The odd-order pure multipolar sources have different number of lobes in forward and backward radiation. The asymmetric superposition of forward and backward scattering induces the formation of a skyrmion in Poynting vector field at the center.

The canonical momentum is directed along the phase gradients of the optical field, so it does not vanish even as the electric and magnetic field vanishes. As such, it does not suffer from the same discontinuities as the Poynting vector and can maintain a continuous deformation to infinity which can generate topological structures.

Lastly, let us consider a superposition of multipoles with the same amplitude in order to determine who dominates in mixed multipole scatterers, since it is difficult to generate a pure octupole compared to a mixture of multipoles in reality. We choose octupole as the highest order multipole component in mixed multipole since octupole source is the simplest source to generate **P** skyrmion and **p** ^**o**^ meron in our system. We observe the formation of a skyrmion in the kinetic momentum field within a radius of 20*λ* (Figure 1(e1)), and a meron in the canonical momentum (Figure 1(e2)). In fact, central features such as the number of reversals in the *z* component of the discussed vector fields are given by the highest order of mixed multipole sources with equal weights.

### 3.2 Helicity tuning

Quasiparticle textures can also be characterized by their helicity *γ* ∈ [−*π*, *π*], which preserves the topological invariants under a global azimuthal rotation, which distinguishes the well-known Néel and Bloch-type skyrmions [47]. To introduce helicity, one must identify a global *U*(1) degree-of-freedom to control. In our case, we have two such degrees-of-freedom – the phase of the Mie coefficients, and the ellipticity of the irradiation field.

**Figure 2:**
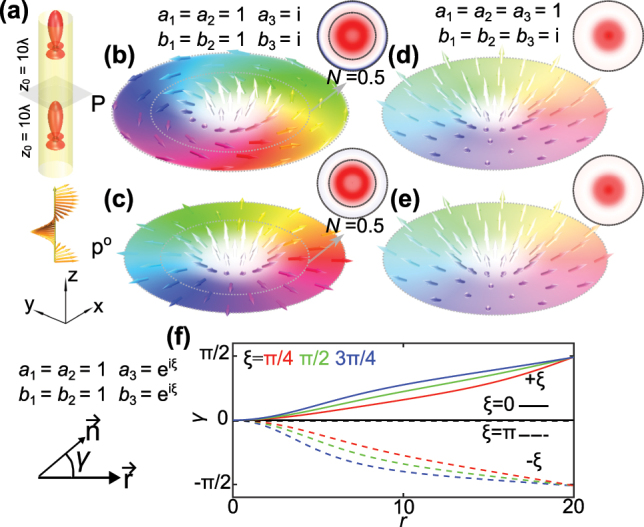
The tunability of the helicity of the topological structure in momentum field. (a) Schematic diagram of the two-particle system, similar to Figure 1. (b–e) The kinetic and canonical momentum distributions, respectively, when (b–c) *a*_1,2_ = *b*_1,2_ = 1 and *a*_3_ = *b*_3_ = *i*, and (d–e) *a*_1,2,3_ = 1 and *b*_1,2,3_ = *i*. (f) The change of *γ* with respect to *r* for different multipole source with phase difference. *γ* ∈ [−*π*, *π*] represents the angle between the in-plane field vector (Poynting vector here) and the radial position vector. The Supplementary Movies 1 and 2 show the dynamic evolution of the textures of **P** and **p** ^**o**^ with respect to *ξ*.

Keeping the multipole coefficients *a*_1,2_ = *b*_1,2_ = 1, we induce a phase to the octupole coefficient *a*_3_ = *b*_3_ = *i* and change the incident field polarization to circular, to couple both the in phase and the quadrature components of the Mie scattering coefficients (Figure 2(a)). The resulting multipole modes are similar to those shown in Figure 1(e), with the octupole component out of phase.

The resulting scattered fields are shown in Figure 2(b) and (c). Because the polarization of the field does not modify the phase gradient, the canonical momentum remains without helicity. Going beyond this simple example, we show that adjusting the phase difference between the Mie coefficients enables control over the helicity of the Poynting vector topological structure (Figure 2(f)).

Finally, we note that if we introduce phase differences between electric and magnetic multipole components, such as *a*_1,2,3_ = 1, *b*_1,2,3_ = *i*, the Poynting vector and the canonical momentum texture in Figure 2(d) and (e), both host merons that extend to infinity and do not exhibit helicity.

**Figure 3:**
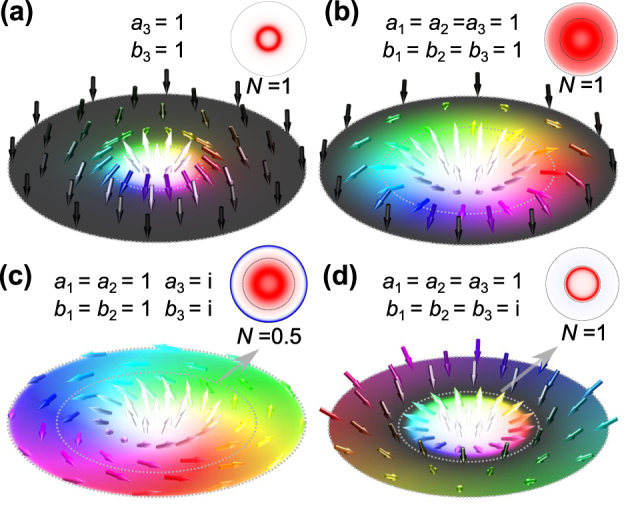
Spin angular momentum textures of scattered fields when the sources are (a) pure octupoles (*a*_3_ = *b*_3_ = 1), (b) mixed multipoles (*a*_1,2,3_ = *b*_1,2,3_ = 1), (c) mixed multipoles with phase difference between different orders (*a*_1,2_ = *b*_1,2_ = 1, *a*_3_ = *b*_3_ = *i*) and (d) mixed multipoles with phase difference between electric and magnetic components (*a*_1,2,3_ = 1, *b*_1,2,3_ = *i*).

In the following, we elucidate how helicity influences angular momentum textures, which is a topic that has garnered significant research interest recently [22], [24], [25]. Nontrivial SAM distributions also arise in our toy system (Figure 3), which originates from SAM of incident light. It is not necessarily related to the phase differences of the Mie coefficient. When the incident light is circularly polarized $E_{\mathrm{inc}}=E_{0}{\text{e}}^{\text{i}\mathrm{kz}}\left(\hat{x}+i\hat{y}\right)$ , for the multipole source *a*_3_ = *b*_3_ = 1, *a*_1,2,3_ = *b*_1,2,3_ = 1 and *a*_1,2_ = *b*_1,2_ = 1, *a*_3_ = *b*_3_ = *i*, the corresponding SAM textures are nontrivial (Figure 3(a)–(c)). Besides, SAM topological textures are the same to **P** topological texture for above three situations, which can be proven by Figure S4 in Supplementary Section V and Figure 3. This phenomenon is common for the first Kerker condition *a*_*n*_ = *b*_*n*_ [46]. For Kerker scattering, the system restores the EM duality symmetry, preserving conservation of angular momentum along Poynting vector direction [48], [49]. When we introduce a phase difference between electric and magnetic components of multipole sources, breaking the Kerker condition, such as *a*_1,2,3_ = 1, *b*_1,2,3_ = *i*, the **S** texture and **P** texture are different. As shown in Figure 3(d), a skyrmion is formed within *r* = 10*λ* in examined plane, which is different to Figure 2(d).

The similarity between Poynting vector texture and SAM texture of multipole scattering field also provides a potential tool for sensing SAM fields, enabling experimental determination of topological quasiparticles. This technique, which uses nanoparticles as probes based on their scattering properties, has been applied in several experiments [50], [51].

### 3.3 Topological stability

**Figure 4:**
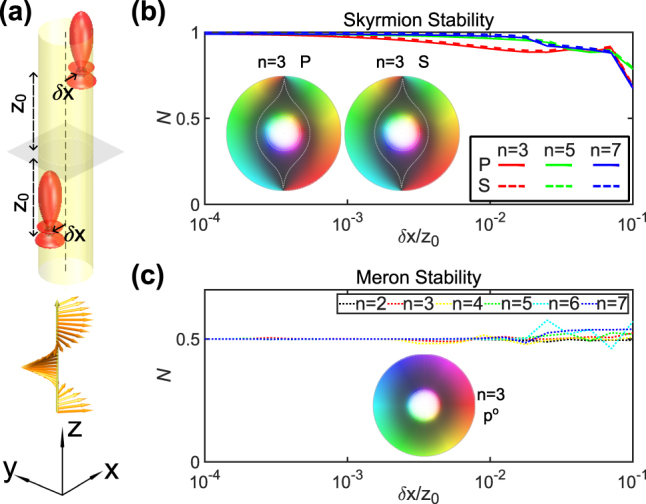
Topological stability of skyrmion and meron. (a) Schematic diagram of two-particle system with positional disturbance *δx*. (b) Topological charge numbers of **P** and **S** textures. (c) Topological charge number of canonical momentum field **p** ^**o**^ with respect to *δx*. The insets show the textures when *δx* = 0.01*z*_0_, where *n* refers to the Mie coefficient order.

Topological protection provides stability to skyrmionic structures. The topological invariant *N* is expected to remain unperturbed under smooth continuous deformations of the physical domain. We demonstrate this by a transverse translation of the multipoles (Figure 4) away from the optical axis. We investigate the change in the topological charge number *N* of the field textures of **P**, **S** and **p** ^**o**^ for pure multipoles (*a*_*n*_ = *b*_*n*_ = 1) upon off-axis translation *δx*. The **P** and **S** skyrmions in Figure 4(b) exhibit a certain degree of topological stability, with their skyrmion number remaining invariant even under strong perturbations to the system. The textures of **P** and **S** when *a*_3_ = *b*_3_ = 1 and *δx* = 0.01*z*_0_ are shown in the illustrations. The outer boundaries of skyrmions are distorted to spindle curves, which reduces their stability slightly compared to merons in **p** ^**o**^ fields shown in Figure 4(c), as their boundary remains in a circular shape. Besides, for **P** and **S** texture, the inner merons within the inner circle in illustrations of Figure 4(b) are also stable. The nontrivial topological structure of **P**, **S** and **p** ^**o**^ were formed based on the directivity, especially the strong forward scattering. For multipoles, the higher the order is, the stronger the forward scattering is, which explains why **P** and **S** skyrmions are more stable when the source orders are higher. In addition, the meron is footed on the strongest forward scattering at center, whereas the boundary of skyrmion depends on first-order scattering outside the center, which is smaller compared to forward scattering.

## 4 Conclusion and discussion

In conclusion, we have shown the generation of skyrmions in a new domain of optical fields, namely the canonical momentum fields, and how they differ from previous work on Poynting fields. As the canonical momentum is a more direct descriptor of the actual motion of matter driven by light, the canonical momentum skyrmions offer strong theoretical bases for understanding and exploiting topologically nontrivial light–matter interactions. We highlight the difference in nontrivial textures in Mie scattering systems, and demonstrated how tuning various Mie coefficients can modify the properties of these topological quasiparticles. In our system, **p** ^**o**^ merons are more stable than **P** and spin angular momentum **S** skyrmions when the scattering particles shift away from the optical axis. Higher-order multipole sources provide more stability for skyrmions. In the future, research on topological quasiparticles could be extended to multipoles in other scattering systems such as metasurfaces [44], [45], [52]. Our work opens the door to possible future studies on momentum-based topological structures in electromagnetic fields and topological nontrivial light–matter interaction.

## Supplementary Material

Supplementary Material Details

## References

[1] Skyrme T. H. R. (1962). A unified field theory of mesons and baryons. *Nucl. Phys.*.

[2] Al Khawaja U., Stoof H. (2001). Skyrmions in a ferromagnetic Bose-Einstein condensate. *Nature*.

[3] Ruostekoski J., Anglin J. (2001). Creating vortex rings and three-dimensional skyrmions in Bose-Einstein condensates. *Phys. Rev. Lett.*.

[4] Fukuda J.-I., Žumer S. (2011). Quasi-two-dimensional skyrmion lattices in a chiral nematic liquid crystal. *Nat. Commun.*.

[5] Duzgun A., Nisoli C. (2021). Skyrmion spin ice in liquid crystals. *Phys. Rev. Lett.*.

[6] Tai J.-S. B., Hess A. J., Wu J.-S., Smalyukh I. I. (2024). Field-controlled dynamics of skyrmions and monopoles. *Sci. Adv.*.

[7] Nagaosa N., Tokura Y. (2013). Topological properties and dynamics of magnetic skyrmions. *Nat. Nanotechnol.*.

[8] Bogdanov A. N., Panagopoulos C. (2020). Physical foundations and basic properties of magnetic skyrmions. *Nat. Rev. Phys.*.

[9] Fert A., Reyren N., Cros V. (2017). Magnetic skyrmions: advances in physics and potential applications. *Nat. Rev. Mater.*.

[10] Han L. (2022). High-density switchable skyrmion-like polar nanodomains integrated on silicon. *Nature*.

[11] Chen S. (2024). All-electrical skyrmionic magnetic tunnel junction. *Nature*.

[12] Shen Y., Zhang Q., Shi P., Du L., Yuan X., Zayats A. V. (2024). Optical skyrmions and other topological quasiparticles of light. *Nat. Photonics*.

[13] Shen Y., Wang H., Fan S. (2025). Free-space topological optical textures: tutorial. *Adv. Opt. Photonics*.

[14] Lei X., Du L., Yuan X., Zayats A. V. (2021). Optical spin–orbit coupling in the presence of magnetization: photonic skyrmion interaction with magnetic domains. *Nanophotonics*.

[15] Yang A. (2023). Spin-manipulated photonic skyrmion-pair for pico-metric displacement sensing. *Advanced Science*.

[16] Yang A. (2025). Optical skyrmions: from fundamental to applications. *J. Opt.*.

[17] Tsesses S., Ostrovsky E., Cohen K., Gjonaj B., Lindner N., Bartal G. (2018). Optical skyrmion lattice in evanescent electromagnetic fields. *Science*.

[18] Davis T. J., Janoschka D., Dreher P., Frank B., zu Heringdorf F.-J. M., Giessen H. (2020). Ultrafast vector imaging of plasmonic skyrmion dynamics with deep subwavelength resolution. *Science*.

[19] Shen Y., Hou Y., Papasimakis N., Zheludev N. I. (2021). Supertoroidal light pulses as electromagnetic skyrmions propagating in free space. *Nat. Commun.*.

[20] Wang R. (2024). Observation of resilient propagation and free-space skyrmions in toroidal electromagnetic pulses. *Applied Physics Reviews*.

[21] Shen Y., Papasimakis N., Zheludev N. I. (2024). Nondiffracting supertoroidal pulses and optical “Kármán vortex streets”. *Nat. Commun.*.

[22] Du L., Yang A., Zayats A. V., Yuan X. (2019). Deep-subwavelength features of photonic skyrmions in a confined electromagnetic field with orbital angular momentum. *Nat. Phys.*.

[23] Dai Y. (2020). Plasmonic topological quasiparticle on the nanometre and femtosecond scales. *Nature*.

[24] Guo C., Xiao M., Guo Y., Yuan L., Fan S. (2020). Meron spin textures in momentum space. *Phys. Rev. Lett.*.

[25] Lin M., Du L., Yuan X. (2022). Photonic pseudospin skyrmion in momentum space. *IEEE Photonics J.*.

[26] Gao S., Speirits F. C., Castellucci F., Franke-Arnold S., Barnett S. M., Götte J. B. (2020). Paraxial skyrmionic beams. *Phys. Rev. A*.

[27] Shen Y., Martínez E. C., Rosales-Guzmán C. (2022). Generation of optical skyrmions with tunable topological textures. *ACS Photonics*.

[28] Sugic D. (2021). Particle-like topologies in light. *Nat. Commun.*.

[29] Wang S. (2024). Topological structures of energy flow: poynting vector skyrmions. *Phys. Rev. Lett.*.

[30] Stratton J. A. (2015). *Electromagnetic Theory*.

[31] Poynting J. H. (1884). Xv. on the transfer of energy in the electromagnetic field. *Philos. Trans. R. Soc. London*.

[32] Gough W. (1982). Poynting in the wrong direction?. *Eur. J. Phys.*.

[33] Minkowski H. (1910). Die grundgleichungen für die elektromagnetischen vorgänge in bewegten körpern. *Math. Ann.*.

[34] Ibrahim I. (1909). Zur elektrodynamik bewegter körper. *Ann. Phys.*.

[35] Abraham M. (1910). On minkowski’s electrodynamics. *Rend. Circ. Mat. Palermo*.

[36] Ghosh B., Daniel A., Gorzkowski B., Bekshaev A. Y., Lapkiewicz R., Bliokh K. Y. (2024). Canonical and poynting currents in propagation and diffraction of structured light: tutorial. *JOSA B*.

[37] Baxter C., Babiker M., Loudon R. (1993). Canonical approach to photon pressure. *Phys. Rev. A*.

[38] Afanasev A., Carlson C. E., Mukherjee A. (2022). Superkicks and the photon angular and linear momentum density. *Phys. Rev. A*.

[39] Barnett S. M., Berry M. (2013). Superweak momentum transfer near optical vortices. *J. Opt.*.

[40] Lu C. (2023). Nanoparticle deep-subwavelength dynamics empowered by optical meron–antimeron topology. *Nano Lett.*.

[41] Yang L.-P., Khosravi F., Jacob Z. (2022). Quantum field theory for spin operator of the photon. *Phys. Rev. Res.*.

[42] Yang Q. (2020). Mie-resonant membrane huygens’ metasurfaces. *Adv. Funct. Mater.*.

[43] Sugimoto H., Fujii M. (2021). Colloidal Mie resonators for all-dielectric metaoptics. *Adv. Photonics Res.*.

[44] Allayarov I., Evlyukhin A. B., Calà Lesina A. (2024). Multiresonant all-dielectric metasurfaces based on high-order multipole coupling in the visible. *Opt. Express*.

[45] Terekhov P. D., Babicheva V. E., Baryshnikova K. V., Shalin A. S., Karabchevsky A., Evlyukhin A. B. (2019). Multipole analysis of dielectric metasurfaces composed of nonspherical nanoparticles and lattice invisibility effect. *Phys. Rev. B*.

[46] Kerker M., Wang D.-S., Giles C. (1983). Electromagnetic scattering by magnetic spheres. *JOSA*.

[47] Zhang Q., Xie Z., Du L., Shi P., Yuan X. (2021). Bloch-type photonic skyrmions in optical chiral multilayers. *Phys. Rev. Res.*.

[48] Fernandez-Corbaton I., Zambrana-Puyalto X., Tischler N., Vidal X., Juan M. L., Molina-Terriza G. (2013). Electromagnetic duality symmetry and helicity conservation for the¡? format?¿ macroscopic Maxwell’s equations. *Phys. Rev. Lett.*.

[49] Zambrana-Puyalto X., Fernandez-Corbaton I., Juan M., Vidal X., Molina-Terriza G. (2013). Duality symmetry and Kerker conditions. *Opt. Lett.*.

[50] Eismann J. (2021). Transverse spinning of unpolarized light. *Nat. Photonics*.

[51] Banzer P., Peschel U., Quabis S., Leuchs G. (2010). On the experimental investigation of the electric and magnetic response of a single nano-structure. *Opt. Express*.

[52] Babicheva V. E., Evlyukhin A. B. (2021). Multipole lattice effects in high refractive index metasurfaces. *J. Appl. Phys.*.

