# Supplementary material for: Topological momentum skyrmions in Mie scattering fields
Source: Nanophotonics. 2025 May 5;14(12):2211–7. doi: 10.1515/nanoph-2025-0071 (PMC12147548; doi:10.1515/nanoph-2025-0071)
Supplement: Supplementary file 1 — Supplementary Material Details [file j_nanoph-2025-0071_suppl_001.pdf]

Supplementary Information of
TOPOLOGICAL MOMENTUM SKYRMIONS IN MIE SCATTERING FIELDS

Peiyang Chen,[1,2,3] Kai Xiang Lee,[1,4] Tim Colin Meiler,[1,5] and Yijie Shen[1,6]

[1] *Centre for Disruptive Photonic Technologies, School of Physical and Mathematical Sciences, Nanyang Technological University, Singapore 637371, Singapore.*
[2] *School of Physics and Astronomy, Shanghai Jiao Tong University, Shanghai, 200240, China.*
[3] *Zhiyuan College, Shanghai Jiao Tong University, Shanghai, 200240, China.*
[4] *Centre for Quantum Technologies, National University of Singapore, Singapore 117543, Singapore.*
[5] *Institute of Materials Research and Engineering, A*STAR (Agency for Science Technology and Research), Singapore 138634, Singapore*
[6] *School of Electrical and Electronic Engineering, Nanyang Technological University, Singapore 639798, Singapore.*

* yijie.shen@ntu.edu.sg

I. MIE SCATTERING THEORY

Mie solution to Maxwell equations describes the scattering of an electromagnetic plane wave by a homogeneous sphere. [1] Consider a linearly-polarized (in x) plane wave whose wave vector $\mathbf{k} = k\mathbf{z}$ is along z-direction, with electric field given by $\mathbf{E_{inc}} = E_0 e^{ikz}\hat{\mathbf{x}}$. The scattered electric and magnetic fields can be written in terms of a vector harmonic expansion

$$\mathbf{E_s} = \sum_{n=1}^{\infty} E_n [ia_n \mathbf{N}_{e1n}^{(3)}(k,\mathbf{r}) - b_n \mathbf{M}_{o1n}^{(3)}(k,\mathbf{r})] \tag{1a}$$

$$\mathbf{H_s} = \frac{k}{\omega\mu} \sum_{n=1}^{\infty} E_n [a_n \mathbf{M}_{e1n}^{(3)}(k,\mathbf{r}) + ib_n \mathbf{N}_{o1n}^{(3)}(k,\mathbf{r})] \tag{1b}$$

Here, $\mathbf{M}_{\substack{e\\o}mn}$ and $\mathbf{N}_{\substack{e\\o}mn}$ are the electric and magnetic vectorial spherical harmonics. The subscripts label the even (e) or odd (o) harmonics, $m = 1$ and n labels the order and the degree of the harmonic. n also indexes the order of the multipole in the expansion. The superscript (3) indicates that the radial part of the generating functions are spherical Hankel functions of the first kind. The electric field contribution for each multipole is given by $E_n = \frac{i^n E_0(2n+1)}{n(n+1)}$. The Mie coefficients a_n and b_n depend on the size , shape and material of the illuminated particle.

$$a_n(\omega) = \frac{\mu m_1^2 [\rho j_n(\rho)]' j_n(\rho_1) - \mu_1 m^2 [\rho_1 j_n(\rho_1)]' j_n(\rho)}{\mu m_1^2 [\rho h_n(\rho)]' j_n(\rho_1) - \mu_1 m^2 [\rho_1 j_n(\rho_1)]' h_n(\rho)} \tag{2a}$$

$$a_n(\omega) = \frac{\mu_1 [\rho j_n(\rho)]' j_n(\rho_1) - \mu [\rho_1 j_n(\rho_1)]' j_n(\rho)}{\mu_1 [\rho h_n(\rho)]' j_n(\rho_1) - \mu [\rho_1 j_n(\rho_1)]' h_n(\rho)} \tag{2b}$$

Here, μ and μ_1 are magnetic permeability of the medium and the particle. j_n and h_n represent the spherical functions of Bessel and Hankel of the first kind, respectively. $\rho = kR$ and $\rho_1 = k_1 R$ with R being the radius of the sphere. $k = \frac{\omega}{c} m$ is the wave vector outside the particle and $k_1 = \frac{\omega}{c} m_1$ is the wave vector in the medium from the particle material, m and m_1 are the refractive indices of the medium and the particle.

II. TOPOLOGICAL MOMENTUM TEXTURE IN HIGHER ORDER MULTIPOLAR FIELD

We show the momentum texture in higher order multipolar fields to validate the characteristics of momentum textures in the scattering fields of even-order and odd-order multipoles mentioned in the main text. As shown in FIG. S1, the kinetic momentum field has a discontinuity around the most central region due to vanishing electric and magnetic fields and a topological invariant cannot be quantified for even-ordered multipolar field e.g. ($a_4 = b_4 = 1$) and ($a_6 = b_6 = 1$), while kinetic momentum skyrmions are realized for the pure odd-ordered multipoles except dipoles, e.g. ($a_5 = b_5 = 1$) and ($a_7 = b_7 = 1$).

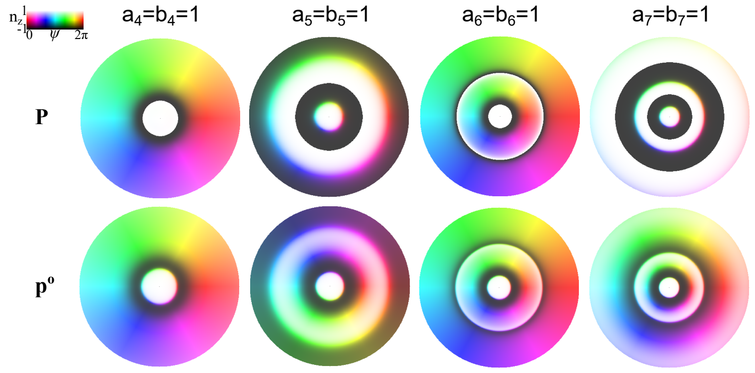

FIG. S1. **Momentum textures in higher order multipolar fields**

III. BOUNDARY DEFINITION

The ideal boundaries of skyrmions and merons can be theoretically determined based on the z-components of the vectors: at the boundary, the z-component is -1 for skyrmions and 0 for merons. Furthermore, we ensure that the values of $\mathbf{P}$ or $\mathbf{p^o}$ at the boundaries are sufficiently large to be measurable. We calculated $|\mathbf{P}|/\max|\mathbf{P}|$ and $|\mathbf{p^o}|/\max|\mathbf{p^o}|$ through numerical simulation, setting 0.1% as a threshold value. This threshold implies that the magnitudes of normalized $\mathbf{P}$ or $\mathbf{p^o}$ above 0.1% can be simultaneously detected, which satisfies contrasts in most experimental conditions.

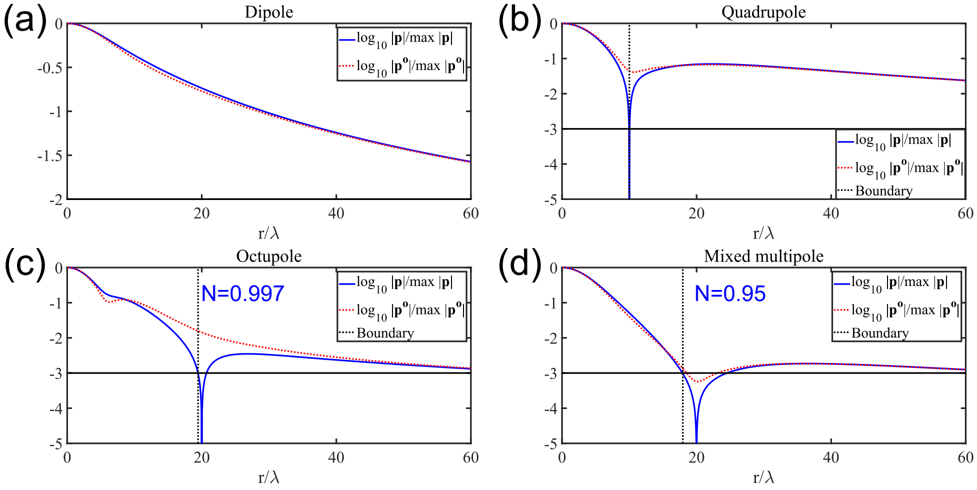

FIG. S2. **Boundary definition of topological quasiparticles. (a-d)** The change of the magnitudes of normalized $\mathbf{P}$ or $\mathbf{p^o}$ with respect to r in logarithmic coordinates. The black dashed lines represent the first radii where $|\mathbf{P}|/\max|\mathbf{P}| = 0.1\%$.

For four typical multipole sources, their corresponding merons' boundaries are easy to define since $|\mathbf{p}|$ and $|\mathbf{p^o}|$ are significantly larger than zero at merons' boundaries, while skyrmions' boundaries are harder to detect. For octupole sources, the skyrmion number N within the boundary is 0.997, as shown in FIG. S2(c). For the mixed multipole, the skyrmion number N within the boundary is 0.95, as shown in FIG. S2(d). This small error demonstrates that we can detect the presence of skyrmions at a detection threshold of 0.1%.

IV. TUNING THE HELICITY

To further illustrate that we can control the helicity of the momentum texture by adjusting the phase difference of the multipole sources, we use animations to demonstrate how the $\mathbf{P}$ and $\mathbf{p^o}$ textures change with the phase difference of the dipole sources, as shown in FIG. S3. Same to the conclusion in the main text, the $\mathbf{P}$ textures have helicity, while the $\mathbf{p^o}$ textures do not. It is worth noting that the change of $\mathbf{P}$ texture with respect to ξ is not azimuth inversion symmetric. For example, when $\xi = \pi/3$ and $\xi = -\pi/3$, the corresponding textures are not inversion symmetric in the azimuth angle. The asymmetry originates from the chirality of incident light.

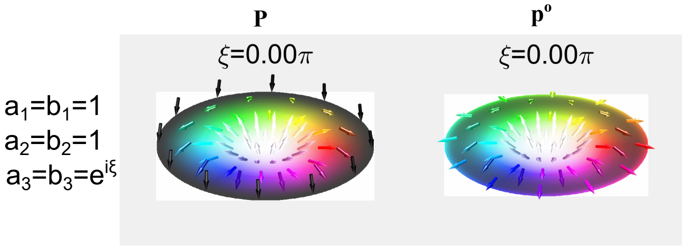

FIG. S3. **Tunability of helicity.** Please see **Supplementary Movie 1** and **Movie 2** for $\mathbf{P}$ texture animation and $\mathbf{p^o}$ texture animation. The change of $\mathbf{P}$ and $\mathbf{p^o}$ textures with respect to phase difference ξ of the multipole sources $a_{1,2} = b_{1,2} = 1 \quad a_3 = b_3 = e^{i\xi}$ when the incident light is circularly polarized $\mathbf{E_{inc}} = E_0 e^{ikz}(\hat{\mathbf{x}} + i\hat{\mathbf{y}})$

V. THE SIMILARITY BETWEEN SAM AND POYNTING VECTOR TEXTURE

When the incident light is circularly polarized $\mathbf{E_{inc}} = E_0 e^{ikz}(\hat{\mathbf{x}} + i\hat{\mathbf{y}})$, kinetic momentum textures are collinear with SAM texture for the multipole source $a_3 = b_3 = 1$, $a_{1,2,3} = b_{1,2,3} = 1$ and $a_{1,2} = b_{1,2} = 1, a_3 = a_4 = i$, respectively, as shown in FIG. S4

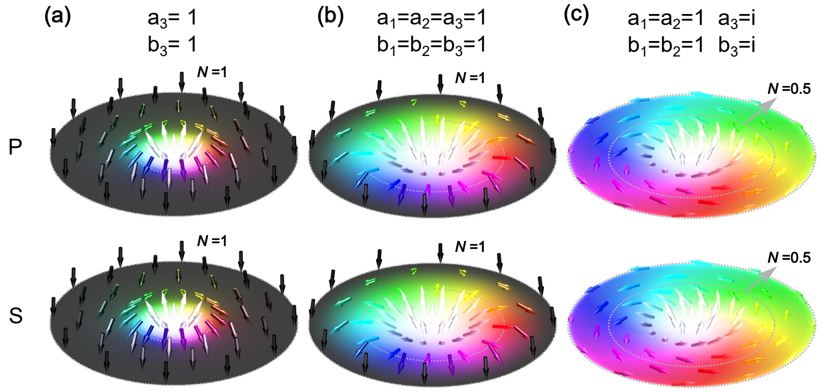

FIG. S4. **The textures of Poynting vector and SAM in multipole scattered field** when the multipole sources are **(a)** pure octupole ($a_3 = b_3 = 1$), **(b)** mixed multipole without phase difference ($a_{1,2,3} = b_{1,2,3} = 1$), **(c)** mixed multipole with phase difference between different orders ($a_{1,2} = b_{1,2} = 1 \quad a_3 = b_3 = i$)

[1] C. F. Bohren and D. R. Huffman, *Absorption and scattering of light by small particles* (John Wiley & Sons, 2008).
